# Retraction: The Gastroprotective Effect of *Vitex pubescens* Leaf Extract against Ethanol-Provoked Gastric Mucosal Damage in Sprague-Dawley Rats

**DOI:** 10.1371/journal.pone.0294006

**Published:** 2023-11-10

**Authors:** The PLOS ONE Editors

Following the publication and correction of this article [1, 2], concerns were raised regarding reuse of results presented in presented in Figs 9 and 10. Specifically,

- Fig 9, the following panels appear to partially overlap, despite being used to represent different experimental conditions:
  ○ Panel G4 and panel G5
  ○ Panel G7 of this article [1] and Fig 6d of [3, retracted in 4]* when rotated
- Fig 10, the following panels appear to fully or partially overlap, despite being used to represent different experimental conditions:
  ○ Panel G1 of this article [1] and Fig 5A of [5]*
  ○ Panel G3 of this article [1] and Fig 10E of [6]
  ○ Panel G5 of this article [1] and Fig 10D of [6]
  ○ Panel G7 of this article [1] and Fig 10C of [6]

Some of the similar (or reused) images were used to represent controls in these articles, but different methodological details were reported for the indicated experiments in the articles’ Materials and Methods sections. Given the nature and extent of the issues, the *PLOS ONE* Editors are concerned about the reliability of data management and/or reporting for this study [1].

A co-author on the article responded that they were unable to retrieve the original data underlying this study, and requested the retraction of the article.

In light of the above concerns, the *PLOS ONE* Editors retract this article.

Some figure panels discussed above appear to report previously published material that are offered under a CC BY license, but the original articles were not attributed in [1]. For these images, the * by the citation, above, marks the oldest publication of the image of which PLOS is aware.

The Fig 10 panel G1 results report material from [5], published in 2015 by Ibrahim et al, under a CC-BY-NC 3.0 license. Due to restrictions that apply to the original article’s license, this figure is excluded from the PLOS article’s [1] CC BY 4.0 license. At the time of retraction, the article [1] was republished to note this exclusion in the Fig 10 legend and the article’s copyright statement.

SMN and MAA agreed with the retraction. MH responded but expressed neither agreement nor disagreement with the editorial decision. NSAW, MFH, SMD, DAB, SMS, ER, HK, RA, AHSA, and HMA either did not respond directly or could not be reached. SMN apologizes for the issues with the published article. MAA stands by the article’s findings.
